# Understanding Resistance Among Survivors of Domestic Violence and Abuse: A Co-Produced Study in the United Kingdom

**DOI:** 10.1177/10778012241309366

**Published:** 2025-01-12

**Authors:** Lucille Kelsall-Knight, Jacky Mulveen, Anna Clarke, Molly Cripps, Caroline Bradbury-Jones

**Affiliations:** 1 Department of Nursing and Midwifery, University of Birmingham, Birmingham, UK; 2 (WE:ARE) Domestic Violence Service, Birmingham, UK

**Keywords:** art, domestic violence, strategies, survivorship, women

## Abstract

Domestic violence and abuse (DVA) is a global problem that affects approximately one in four women in their lifetime. An area of unexplored research is how women use a range of strategies to honor resistance, manage the risk, avoid abuse, and cope with the pain of DVA. Art-based methodology and interpretative phenomenological analysis approaches were used which determined seven strategies employed by women in order to survive DVA: apparent compliance; maintaining a sense of self; knowing boundaries; keeping hope in sight; imagining freedom; degrees of rebellion; and denial. This created a conceptual model for understanding DVA survivorship (The SEED Model).

Domestic violence and abuse (DVA) is understood to be any occurrence or pattern of behavioral control, violence, or abuse, experienced by those who are or have been intimate partners or family members (United Nations, 2021). This may include but is not limited to, physical, emotional, psychological, sexual, and financial abuse. Intimate partner violence (IPV), a closely rated concept that is often used interchangeably with DVA, is abuse perpetrated by an intimate partner or former partner (World Health Organization, 2021).

The World Health Organization (2021) estimated that globally, 27% of women aged 15–49 years have suffered physical and/or sexual IPV at least once in their lifetime. For the year ending March 2023, the crime survey for England and Wales showed that 4.8% of adults aged 16–59 years (2.3 million people) had experienced DVA in the past year, within which, the victim was female in 73.5% of cases (Office for National Statistics, 2023). The frequency and severity of abuse perpetrated against women manifests as a result of interpersonal and structural factors, associated with power relations between women and men (McFeely, 2014). While heterosexual cisgender women are disproportionately affected by DVA in comparison to other groups, Donovan and Barnes (2017) argue that dominant, heteronormative discourses around female victims and male perpetrators minimize the diversity of abusive experiences. Accepting these viewpoints, this article focuses on women who have experienced DVA, while not focusing exclusively on male perpetration.

Levels of depression, anxiety, and post-traumatic stress disorder (PTSD) are elevated among survivors (Chandan et al., 2019; Ferrari et al., 2016). Degradation of self-esteem and detachment from support networks causes reduced self-efficacy and loneliness (Lin-Roark et al., 2015). In many women who have children, lingering feelings of attachment and loss of the wife-mother role contribute to a sense of identity disruption (Estrellado & Loh, 2016). Psychological responses to the aforementioned stressors are variable. Sufficient availability of coping resources can improve separation outcomes for survivors; coping resources include self-efficacy, material resources, and structural and interpersonal support (Sauber & O’Brien, 2017).

As regards terminology, there is an argument that reference to individuals who have experienced abuse, as “victims” perpetuates stigmatization, depicting individuals as helpless (Dunn, 2005). On the other hand, the term “survivor” has positive connotations and instills a sense of empowerment. In this paper, we use the term “survivor” with reference to women who have experienced DVA. Previous research conducted by (Heywood et al., 2019) has brought the notion of “thrivership” to the fore in the context of DVA. Thrivership is seen as a fluid journey of self-discovery and is a concept that is characterized by a positive outlook and looking to the future, improved health and well-being, a reclamation of the self, and a new social network (Heywood et al., 2019). Such research has offered a useful lens through which DVA survivorship and thrivership can be understood (and the nuanced differences between these concepts). In a similar vein, previous research has explored the place of agency (Mannell et al., 2016; Pells et al., 2016) and resistance (Black et al., 2020; Mkandawire-Valhmu et al., 2016; Morgan & Coombes, 2016). However, a detailed explication is missing from contemporary literature as regards how women strategize as part of resistance. We do not fully understand the range of strategies women use to honor resistance, manage the risk, avoid abuse, and cope with the pain, all while living under a regime of power and control. This study sought to address this gap through an in-depth, qualitative exploration of how women used a range of strategies to survive DVA what these hold for themselves as survivors, and the services that support them.

## Methods

### *Research Design and Procedures*

This study adopted a flexible, participatory, and creative, qualitative design. Art methodology was employed, alongside focus group interviews, as a way of using a representational form of inquiry regarding the participant’s subjective experiences of a sensitive topic. Focus group interviews were conducted either face-to-face or via Zoom by [LKK and CBJ] with the participants. The participants had created a piece of artwork, prior to the focus group that detailed their survivorship of DVA, with the intention to use the artwork as the focus of discussion. Some participants created the artwork specifically for the focus group (e.g., taking a photograph of items that were used to escape the abuser), whereas other participants had created artwork as part of the DVA program and they brought this to the focus group to discuss how it detailed their resistance to DVA and survivorship. All groups were audio recorded and transcribed verbatim. Data were analyzed using Interpretative Phenomenological Analysis (IPA). Two members of the team undertook the initial analysis [AC and MC] and the tentative themes were shared and discussed with the rest of the authors at a face-to-face analytical decision-making session held in July 2022. By the end of the two-hour session, the themes had been revised and agreed across the team.

IPA has gained increasing momentum in recent years within health and social sciences, due in part to its ability to interpret and understand human experiences, especially those that are concerned with emotionally charged, complex topics (McInally & Gray-Brunton, 2021). The theoretical foundation of IPA lies in phenomenology, which explores individuals’ perceptions and considers how individuals reflect on significant life experiences (Smith et al., 2009). Researchers who undertake IPA acknowledge that experience is subjective and open to interpretation, only accessible through interpretation (Biggerstaff & Thompson, 2008). We considered it a fitting analytical framework for our study because of its inherent flexibility and because of its focus on the subjective experience of individuals, as the data analyst [AC and MC] in this study played a central role in sense-making regarding the participant’s individual experiences.

### *Ethical Approval*

Ethical approval was obtained from the University of Birmingham in 2021. ERN_21-0812. The study adhered to COnsolidate criteria for REporting Qualitative research (COREQ) guidance (Tong et al., 2007).

### *Patient and Public Involvement*

The gatekeeper to the DVA organization was pivotal in contributing to the ethics application and study design. They ensured the correct terminology was used and that the design was appropriate and safe. In addition, they confirmed there was support in place for any of the participants who may disclose upsetting information and require additional support. Study participants were involved in co-designing the study and its outputs, by means of constructing their artwork and by influencing the need for focus groups to discuss their artwork. The participants attended a creative day (in July 2022) whereby sense-checking of the emergent findings was undertaken. It was at this event that the SEED model evolved as a diagrammatic representation to frame the findings. By the end of the three-hour event, the entire team had agreed on the themes derived from the data and a rudimentary form of the SEED model had been co-designed (it was subsequently modified and agreed in the form presented in this article).

### *Participant Recruitment and Procedures*

The participants were purposively recruited through an open invitation distributed to women who were currently accessing a free-of-charge group service offered to women who have experienced DVA, based in the West Midlands region of the United Kingdom. The group offers a unique selection of programs to women from across the region. The core areas of the program focused on inclusive and exploratory group work that aims to build the women's self-esteem, empower them, and enable them to build positive relationships moving forward, after the abuse. Central to the ideology behind the project is that women who experience DVA can be empowered to thrive after abuse. Participants were eligible to take part in the study if they were aged 18 years and older and had survived DVA. The participants were no longer living in an abusive relationship. Participant information sheets were sent to women who expressed an interest in participating in the research project. The primary author then made contact with the interested participants via a group online meeting to provide more detailed information about the study. None of the participants were known to the study investigators, but some of the participants were known to one another due to their involvement in the DVA program. All participants were offered a £20 shopping voucher as an incentive for their participation. Sixteen women participated in the study, with varying employment backgrounds including being stay-at-home parents and senior management professionals. The women provided written consent prior to participation, witnessed by the researchers. As part of the consent process, they were aware of their withdrawal rights and the necessary matters of anonymity, confidentiality, and protection of data.

### *Data Collection*

The focus groups took place either face-to-face or via Zoom in December 2021. The participants chose which medium they preferred to undertake the focus group and they were held at a time that was agreeable to all on three separate dates so that the women could select the most appropriate time and date for them. All the participants signed an informed consent sheet and were interviewed in a focus group by the first and last authors (experienced female qualitative researchers). The primary author, with the gatekeeper of the service provider, was recruited until data saturation was met. Data saturation in qualitative research is the point when new data no longer provides new insights into the research question being asked (Saunders et al., 2018). Data saturation was determined when there was adequate data to develop an understanding of the study phenomenon and further coding was no longer necessary.

Participants were asked to bring the piece of artwork that they had constructed and to share the story behind it, which they felt enabled them to show their route to DVA survival. They were also asked, in a summary of one word, to say what resistance of DVA meant to them. There were six participants in focus group 1 (Zoom) and five participants in focus groups 2 and 3 (face-to-face). The duration of the focus groups ranged from 1 h 40 min to 1 h 58 min. A semistructured interview style was adopted and used flexibly. The term “resistance” was not defined to the group, but instead was a word that was used to facilitate discussion regarding what it meant to the individuals. The interview used the following prompts:

*Talking about resistance*
What does it mean?
How do women resist in situations of DVA?
What actual actions do they take?
(If women want to) How does your creative work today capture your resistance to DVA?
*Talking about coping*
What does it mean?
How do women cope in situations of domestic abuse?
What actual actions do they take?
How do coping and resistance link?

### *Data Analysis*

The transcribed interviews were analyzed by [insert initials following blind peer review] using IPA. The IPA involved: initially listening and re-reading the interviews a number of times alongside looking at the artwork to ensure that a sense of the participants’ accounts was gained. Secondly, emergent themes were identified and connections across the individual focus group transcript were examined. Lastly, a table of themes was created for each transcript and this was integrated into the final analysis. The themes were subjective and open to interpretation (Biggerstaff & Thompson, 2008); therefore these analyses were then presented to the co-authors and participants for review and acceptance.

## Findings

**Figure 1.**
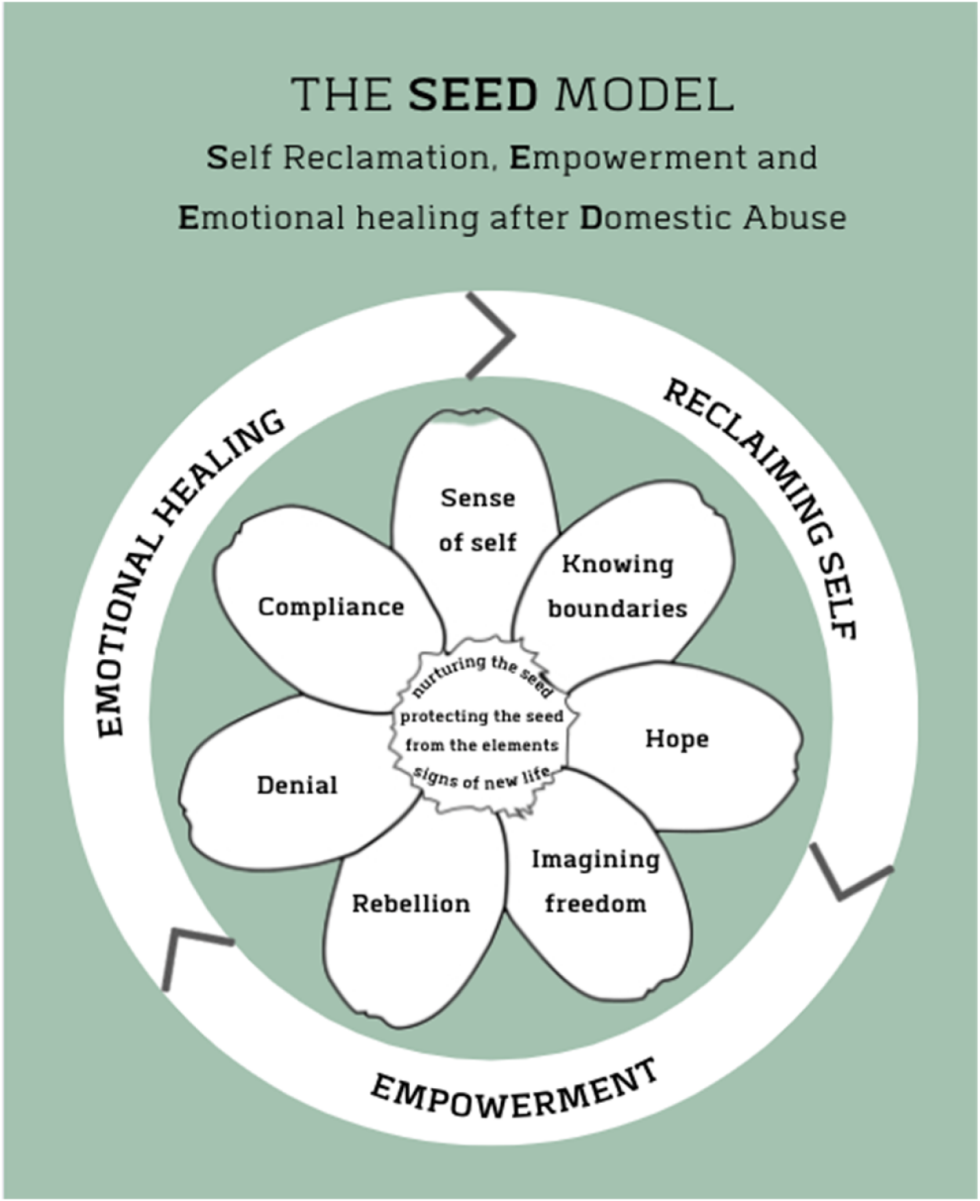
The SEED model (self-reclamation, empowerment, and emotional healing after domestic abuse).

Following analysis, the team and participants agreed that in order to maintain the creative elements of the research project, the analogy of a seed would be used to showcase core concepts of survival in the context of DVA. Three concepts were determined: nurturing the seed, protecting the seed from the elements, and supporting growth. As an extension of the concepts of survival, from the artwork and focus group interviews, we identified seven strategies: compliance, sense of self, knowing boundaries, hope, imagining freedom, rebellion, and denial. Lastly, we found that when women had employed some or all of the seven survival strategies, three outcomes of survival were determined: self-reclamation, empowerment, and emotional healing of DVA. These findings went through several iterations until the whole team was in agreement, and using a participatory approach, were developed in collaboration with the participants. To display the findings visually as a whole, we collectively worked on developing a model. We called this the SEED model, as an acronym for **S**elf-reclamation, **E**mpowerment, and **E**motional healing after **D**omestic abuse. The SEED model is presented in Figure 1, followed by a narrative description of its different elements and participant quotes.

### *Strategy #1: Apparent Compliance*

One strategy that women employed for survival and avoiding abuse, was compliance (or at least an apparent compliance) with the perpetrator. This included obeying demands, avoiding aggravating the perpetrator, remaining silent, and overcompliance in fulfilling demands.

Participant 3: But I knew that one of my coping strategies, as you know, was to be silent, so I planned my way out gradually, so what I did was I thought right, I know that keeping silent is something which suppresses his anger.

Several participants described stopping hobbies either consciously as to avoid conflict or subconsciously as a side effect of constant focus on keeping their partner amiable.

Participant 11 You lose kind of every kind of hobby or thing that you might do, because like you say, everyday your head is constantly thinking about keeping them happy.

Participant 8: I remember there was one top, and it's still in the wardrobe, for some reason I can’t get rid of it, even though I hate it and it reminds me of him, but he’d always go, “oh yeah I really like that.” So, I’d always end up wearing it to please him, even though I hated it.

### *Strategy #2: Maintaining a Sense of Self*

Maintaining a sense of self was a recurring theme across the transcripts. This appeared in a variety of contexts including engaging in hobbies for personal enjoyment. One woman described going to her workshop to make jewelry as a creative escape that enabled her to remind herself of who she was:

Participant 9: Anything creative that I could do, I think that really helped me, sort of thing. It was my way of keeping my sense of self, I think, in any way I could. Urm, so I think going to the workshop it was always like an escape, I got to feel like myself.

Another participant described taking up a new hobby, in contrast to elements of the compliance strategy, by training to run a marathon, this enabled her to have protected time in her running group away from her partner as well as empowering herself through achieving goals:

Participant 6: It felt like my old self because I decided I was going to do something, and he wasn’t going to stop me.

One woman who was a musician described going to work to perform enabled her to maintain a sense of self by both the focus required for the music meaning she could not think about the abuse in addition to finding her identity as a musician.

Participant 10: You’ve got no brain space left to think about how badly you’re being treated. And I can then be myself and get really into the music and think, “this is what I’ve trained to do, this is me, this is how I present myself.”

### *Strategy #3: Knowing Boundaries*

A strategy for many women avoiding abuse was the awareness of boundaries, knowing what would trigger the partner, and what the safe limits were. An example of this is avoiding the perpetrator when they were likely to abuse, this was a strategy that could be used once the survivor was aware of the triggers and able to pre-empt unsafe situations:

Participant 1: I’d stay out for about three hours because I knew he’d calm down then, and I could go back in. So that was my resistance, knowing what could happen, pre-empting what could happen.

Altering behavior was another element of knowing boundaries and has some overlap with compliance, this included avoiding behaving in such a way that was likely to trigger abuse.

Participant 7: You find that you behaved a certain way, when he was, you get to a point where you behave a certain way to stave off comments or behaviour.

### *Strategy #4: Keeping Hope in Sight*

Holding hope for a future for life for themselves without the abuser and in which someone would understand what the individual had been through, and, most importantly, believe them was a common strategy for survival:

Participant 10: [I was thinking that] one day people would believe what went on. Because it was kind of … the gas lighting going on and stuff, no one would ever believe you, because you’re mentally ill. Urm, so yeah, for me it's, I know the truth, someone will believe that someday.

Participant 3: What might happen, it might not happen, so you carry on dreaming you carrying on thinking, everything will be okay, everything will be fine in order for you, to be able to survive, to look after your children.

### *Strategy #5: Imagining Freedom*

In answer to the question of what resistance means to them one participant summarized it as:

Participant 10: It's dreaming I suppose, of the future, without him.

Another recurring theme in this strategy was imagining the death of the abuser, it is important to note this was not expressed as intent to harm the partner, but rather imagining a scenario in which the abuser had died and what that would mean for the individual’s future, and the freedom that would provide.

Participant 1: I was imagining him in an accident and dying and I know that sounds awful, but it was that kind of only hope that you’re going to be free… And I just imagined each night when he went out, that the door was going to knock and it’s going to be the police saying he was dead and I practised how I was going to pretend to be sad. I was not heartless but imagining a better life. You know when you think that’s your only way of hope and being free, it’s resistance isn’t it.

Participant 2: My ex used to snore and he used to do that, where they hold their breath … and I used to think “Oh, please don’t breathe.”

### *Strategy #6: Degrees of Rebellion*

Rebellion was described to be a common strategy for honoring resistance. This occurred in both subtle approaches and in methods in which the victim rebelled with more extreme intent. Fighting back was described as an overt element of rebellion, however, most methods tended to be more subtle such as dressing a certain way when not with the abuser, in rebellion to coercive control of what the individual wore each day, and eating food outside the house that she was forbidden from. Rebellion was a strategy used to cope with abuse that didn’t involve avoiding abuse but rather, doing something despite knowing the potential consequences:

Participant 6: I’m thinking so that would put me into danger, but I still would do them little things—that’s resistance that no, you will not control me in every single way.

Participant 11: He used to insist on the knives and forks, and everything set for breakfast the night before. And he’d do the same before he left for work for tea so I couldn’t use the table to work on. So, I used to put everything away the minute he left the door and get it all out before he came home.

Another element of rebellion came from recording the abuse, in various mediums, to ensure that one day someone would find out the truth either when the survivor was ready to share it or in the case of their death:

Participant 13: I used to write poems and I’d sneak them around the house and stuff, so if I did, you know, be found dead one day, under suspicious circumstances, my poems were basically saying about the abuse.

Participant 15: I must have had over 600 voice memos, because literally I just put my tape recorder in my tights or whatever and you know, everything was recorded.

### *Strategy #7: Denial*

The strategy of denial occurred both as a conscious decision and a subconscious one. Some women had to deny to themselves the situation they were in, to enable them to hope for the future or to protect themselves from the truth of the situation:

Participant 3: If you were to face the reality that I or my child could die. I think it would hinder my ability to be able to feel that I can escape and get to a place of safety.

Participant 1: It's that cognitive dissonance isn’t it, because the situation we’ve been in was so intolerable, we convince ourselves otherwise.

An element of denial was strategies to hide the situation from others, one woman described despite living in her car due to the abuse, she would wear smart clothes for work and present herself as a version of herself before the abuse in order to deny the outside world that anything untoward was happening.

Participant 15: I was homeless at one point, and you know, virtually living in my car, but nobody at work would have known because I had nice clothes from when I bought nice clothes. And you know and, it's that putting an act on, you’re still almost in denial and you don’t want people to know.

### *Outcomes*

In addition to the seven strategies for survival, three outcomes were identified through the IPA. These outcomes were achieved after one or more of the strategies had been used and occurred both during and after the abusive relationship.

### *Outcome #1: Emotional Healing*

Emotional healing was an outcome that mostly occurred after the relationship. One participant, however, described an outcome of keeping hope in sight as the building of resistance. This is because she was able to separate herself emotionally from her partner and begin healing, which enabled her to exit the relationship eventually.

Participant 4: It built the resistance, and it built the resilience. It made me see what I could do when I really have to … it took me many years, but that was the foundation, and that was what was important for me that was the beginning of me moving away from him emotionally and finding you know ways of coping.

Emotional healing after the relationship was experienced by some participants by moving away physically from the place, they were in to then enable emotional healing away from triggers. Others described realizing they were healing emotionally when they were able to do things they had been unable to do during the relationship. These acts were no longer a form of rebellion, but healing.

Participant 8: But every time I do something in the house, like paint a wall, I just, like I take like a little moment to enjoy, like knowing it would piss him off.

### *Outcome #2: Self-Reclamation*

Self-reclamation was an outcome different for the strategy of maintaining a sense of self, as instead of it being a way of protecting oneself in order to resist abuse, it was an outcome of such resistance. Self-reclamation was achieved when the women were able to reclaim elements of who they were before the abuse, in addition to honoring who they had become. One participant who enjoyed designing and making jewelry had designed her own wedding ring. With the relationship ended and going through divorce, she altered the ring to reclaim it, instead of getting rid of it. We understood this to be her way of acknowledging and honoring the fact that she got through that time, demonstrating resistance and self-reclamation.

Some participants described the sense of self-reclamation after the relationship, whereby they had claimed something back of their former self:

Participant 12: I went out with friends and had a really nice afternoon off and in a way, for the first time in so many years I realised that I am starting to get myself back.

### *Outcome #3: Empowerment*

An outcome that often came from degrees of rebellion was empowerment. One participant described empowerment after “rebelling” her husband by fighting back when he was attempting to rape her, as a turning point in the relationship and the moment that she realized her resistance had been growing.

This was also an outcome that came from when participants had used their strategies of resistance to leave the relationship and then in sharing their experience experienced empowerment from this. This empowerment facilitated both self-reclamation and emotional healing.

Participant 5: sharing your story, which is something you’ve been ashamed of … it’s like turning your pain into power somehow like I’m not silenced anymore, and I’ll speak my truth, if I want to.

Participant 7: Almost a sense of, now that we rediscovered who we are and… We’re not going to be invisible.

## Discussion

The nature of this study surrounds personal and sensitive topics, and was devised as a way of exploring, through art, how women use a range of strategies to honor resistance, manage the risk, avoid abuse, and cope with the pain, all while living under a regime of power and control. The language surrounding DVA can drastically change the way that the topic is discussed or the lens it is perceived through. As well as the choice to use “domestic abuse” rather than “domestic violence” to highlight the various types of abuse within intimate relationships, the terms used to describe the individuals involved were also carefully considered. While the terms “victim” and “perpetrator” are supported and necessary for legal purposes (Easteal et al., 2012), they are criticized from a social perspective as they are limiting an individual's own identity and experience. Instead, it is suggested that “person/woman/man who has experienced violence,” and “person/woman/man who has perpetrated violence” are used retrospectively, as this highlights the personal development one can experience after DVA (Fulu et al., 2013). With these criticisms in mind, the participants of the study were invited to comment on the terminology used. The overwhelming response was that the term “survivor” is used in place of “victim,” as the study focuses on the strategies these women used to survive the abusive situations and to encapsulate the resistance displayed. As this study follows an Interpretive Phenomenological Analysis framework, the emphasis was placed on conveying the participant's stories in a way that reflects their experience. In addition, the participants are also referred to as “woman/women,” as they were also keen to place an emphasis on their identity separate from their experiences with DVA.

This study has provided new insights into the ways in which women (and mothers) resist and thrive in the context of living with experiences of DVA. Such understandings are important because children are frequently overlooked in DVA situations in terms of their impacts on them. They may not experience direct violence from the perpetrator and are therefore reduced to “witnesses” of the situation (Callaghan et al., 2015). However, the long-term psychological effects on children who have been exposed to DVA are extensive and often contribute to a cycle of abuse (Stanley et al., 2012). Our study findings have shown how mothers carefully and strategically keep themselves and their children safe by demonstrating an apparent compliance with the perpetrator. Morgan and Coombes (2016) have referred to the notion of “protective mothers,” which is very much supported by the findings of our study. As an act of protection, mothers impose strategies to protect their children from violence at the time of abuse and to promote healing and recovery in the long-term. They do this through strategies such as using key terms with their children to warn them when to remove themselves from a dangerous situation, such as “go to your room.” In addition, mothers will instill hope in their children and offer reassurance that the abuse is not the child's fault (Haight et al., 2007). The “failure to protect” is often an unfair criticism levied against women who have experienced DVA in relation to their children. This oversight of the wider factors affecting a women's ability to physically remove a child from a domestically abusive situation further prevents the blame from being placed on the perpetrator (Humphreys & Absler, 2011). In a seemingly opposite strategy to compliance, women described how they quietly and silently engaged in acts of rebellion, carefully judging boundaries and not “pushing too far.”

This paper has considered the language used surrounding DVA, particularly, the victimization of women who have experienced DVA. The participants of this study have focused on thrivership throughout their healing, shifting the narrative away from the victimization of survivors. The idea that women are beginning to reject the victim identity may be crucial in shifting the perceptions that people hold about domestic abuse survivors (Leisenring, 2006). The victimization of women throughout history is thought to have created a “learned helplessness,” whereby women who have experienced trauma, essentially give up in trying to change their situation (Gondolf & Fisher, 1988). It is important to challenge the narrative of learned helplessness in this context. There are myriad, legitimate barriers to leaving a domestically abusive relationship and rational reasons why women stay (Bradbury-Jones et al., 2014). In challenging the mistaken idea that women are helpless and passive victims of their situations, help services may become more accessible to women in these situations and may challenge victim-blaming beliefs (Cunniff Gilson, 2016).

Most women in our study spoke of hope and imagined freedom and this was critical for them to maintain a sense of self. Women who experience DVA are often questioned on their reason for staying in the relationship. The reasons that women stay with a perpetrator of domestic violence are varied and complex, often rooted in the idea of the patriarchy and the gender roles within relationships (LaViolette & Barnett, 2014). It can take several cycles of attempting to leave and returning to the relationship before a woman is far enough removed from their romantic connection to leave (Baly, 2010). While this psychological perspective is perhaps valid, it is more important to acknowledge the structural issues that make it difficult to leave; facing homelessness, relocation, and financial loss are not appealing options. Moreover, it is known that postseparation is a time of heightened risk for stalking and homicide (Nikupeteri, 2017; Nikupeteri & Laitinen, 2015).

Our study has shown how emotional healing is possible after DVA. This is an important finding because it disrupts deterministic narratives about the inevitability of enduring and life-long “damage” of DVA. While not ignoring the long-term impacts in any way, the study findings highlight how a positive outlook and future are possible following DVA. All of these impacts are recognized in the study as outcomes of DVA. These impacts and outcomes are associated with thrivership, which is a complex concept, and in order for thrivership to occur the three key conditions of provision of safety, sharing the story, and social response must be met (Heywood et al., 2019). Self-reclamation and empowerment in this study were conceptualized as the outcome of the strategies for resistance. Like emotional healing, this is an important finding because it highlights the potential for the well-known, long-term impacts of DVA to be overcome and gives hope to survivors for a reclaimed and empowered sense of self, while also acknowledging the long-term impacts of DVA (Heywood et al., 2019).

## Limitations of This Study

The study had a small number of participants (Biggerstaff & Thompson, 2008), which while not unusual for qualitative research, it should be noted that the experiences of DVA survivors shared in this paper may not be generalizable. However, the study has provided insights into a less explored element of DVA and will extend understanding of the phenomenon and the role of art. The IPA framework requires the researcher to be reflexive throughout the process. The authors [LKK, AC, MC, CBJ] all had knowledge of DVA previous to the study; however, one author [JM] has a long-standing role in a community-based DVA program and may have had an influence on the way the women were able to process their abusive situations. This potential for bias was recognized and to mitigate [LKK and CBJ] both separately obtained consent from the participants and acted as leaders of the focus groups, emphasizing that the participants were free to share as much or as little as they felt appropriate. As this is a qualitative study, we sought to amplify the voices of the participants with lived experience of DVA and to provide more insight into an under-researched area of DVA. This transparency is a shared value among the research team, as the aim throughout has been to raise awareness of the power of resistance in the context of DVA survivorship and thrivership.

## Conclusion

This study has placed a focus on survivorship outcomes and the strategies employed by women while living in an abusive relationship. In keeping with the use of art in the study, the creation of the conceptual SEED model allows the reader to visualize how employment of the seven strategies can lead women to three outcomes of DVA survival: emotional healing; reclaiming self; and empowerment. We hope that the study findings will be useful to DVA services, with particular attention to the key elements detailed in the model, to support women in their recovery. The SEED model is a positive framework that disrupts the negative connotations of victimhood and casts a lens on the power of resistance and the potential for healing, survivorship, and thrivership.
